# *Gamification*: focus on the strategies being implemented in interventions: a systematic review protocol

**DOI:** 10.1186/s13104-019-4139-x

**Published:** 2019-02-22

**Authors:** Sílvia Lopes, Armanda Pereira, Paula Magalhães, André Oliveira, Pedro Rosário

**Affiliations:** School of Psychology, University of Minho, Campus de Gualtar, 4710-057 Braga, Portugal

**Keywords:** Gamification, Intervention, Systematic review, Gamify, Game elements

## Abstract

**Objective:**

Gamification broadly refers to the use of game design elements in non-game contexts with the goal of promoting users’ engagement. Gamification strategies appear as an advantageous tool to increase the motivation and involvement of users. The purpose of this systematic review is to identify studies using gamification strategies in distinct intervention contexts and to describe their impact in each type of intervention. Thus, the focus is on the construct (gamification) rather than on a particular area or population.

**Results:**

To achieve this goal, Scopus, IEEE, Web of Science, MEDLINE, ERIC, and PsycINFO databases will be used to gather data for the systematic review. Both the research and report of results will be based on Cochrane’s recommendations and PRISMA guidelines. Data, including the assessment on the quality of the articles, will be conducted by two members of the team independently. Findings will be reported narratively. The ethical approval is not required as the research does not involve data collection. Findings will be submitted to a peer-review journal and the results will be presented on international congresses. Future reviews could consider investigating particular areas of intervention in which gamification strategies are being employed.

*Trial registration* Systematic Review Registration: PROSPERO CRD42017070508

**Electronic supplementary material:**

The online version of this article (10.1186/s13104-019-4139-x) contains supplementary material, which is available to authorized users.

## Introduction

The term *gamification* was originally coined in 2008 [1] within the digital media industry, and broadly refers to the use of game design elements in *non*-*game contexts* with the goal of promoting users’ engagement [1]. From 2010 onwards, interest in the subject has spread into distinct domains of knowledge (e.g., education [e.g., 2], health [e.g., 3] and business management [e.g., 4], and the term has been referred in scientific publications, academia, publicity, industry and health [e.g., 1, 5–7]. The purposes of implementing gamification in such contexts were manifold, such as binding individuals to the tasks at hand, increasing productivity and financial profit, and increasing overall interest in the task [1, 6].

Aiming to promote engagement and motivation in the task at hand [1, 8], gamification works by transferring specific features of games to systems working in non-traditional gaming contexts (e.g., rehabilitation) [1, 6]. In fact, gamification has been analyzed in light of psychological theories of motivation, such as the theory of intrinsic motivation [e.g., 9]. Accordingly, gamified systems present motivational characteristics, such as immediate feedback, indications regarding individual progress or goal setting attainment (through scores, emblems, competitions), relational support, and social feedback and autonomy (to control avatars, and decision making) [1]. Thus, especially for younger populations, the components of gamification may improve the intervention’s intrinsic interest, promote learning opportunities, enhance the degree and level of participants’ engagement, and foster their behavior and lifestyle [3, 10–12]. This is accomplished by involving users through strategies as follows: setting short, medium, and long terms goals, using challenges to display interest, and providing rewards (e.g., badges) for completing actions or achieving scores within a specific grade range on an activity [13, 14]. For instance, in the educative context, gamification of learning may involve “the use of game elements, including action language, assessment, conflict/challenge, control, environment, game fiction, human interaction, immersion, and rules/goals, to facilitate learning and related outcomes” [15, p. 6].

Unequivocally, the use of game-design elements constitutes a unique opportunity to engage participants in specific activities (e.g., rehabilitation or clinical interventions) [16–18]. Regarding the use of gamification in the context of an intervention, two main categories can be distinguished regarding their purpose: (i) gamification for development, i.e. the process of using gamification to build, for example, a new tool or platform with specific purposes; and (ii) gamification for enhancement, i.e. the practice of gamification to increase an already well-established activity [19]. However, implementing a gamification approach within interventions (e.g., rehabilitation or educational focused) is considered a difficult task, and it is listed within the group of complex interventions [19]. All considered, it is necessary to further understand the phenomena and to help practitioners [19].

Previous reviews on this subject addressed important aspects of gamification strategies in different areas of knowledge (e.g., healthcare) and have highlighted that gamifying experiences translate into improvements in psychological and behavioral outcomes [6]. However, none have enquired about the game components and assessment strategies used or their relationship with the efficacy of the interventions. Besides, embedding gamification strategies into interventions is becoming ubiquitous; therefore, it is critical to systematize available information on which elements are being incorporated, and examine how they add up to interventions-as-usual effectiveness regardless of the purpose of the intervention or of its target population. Despite the significant increase in studies using gamification strategies in different areas, little is known about the effectiveness of the use of gamification [8]. Thus, it is important to further understand the phenomenon in depth. Accordingly, the objective of this qualitative review is to understand the phenomenon of gamification, and its effectiveness, regardless of the domain of the intervention (e.g., health, academic) in which the gamification strategies were employed.

## Main text

The present protocol was developed and submitted by the authors for registration in PROSPERO prior to the beginning of the research. The protocol followed the procedures considered in the writing of systematic reviews, namely Cochrane and PRISMA guidelines. All studies including interventions with gamification were considered for review. For a better understanding, authors considered *intervention* as any action on a given population with a therapeutic purpose and *gamification* as “the use of game design elements in non-game contexts” [1].

This systematic review protocol is in line with the items of the Preferred Networks for Systematic Review Initiatives and Meta-Analysis Protocols (PRISMA-P) [20] (see Additional file 1). The current systematic review will follow the guidelines of the Cochrane Collaboration, namely the search strategy, identification, collection and characterization of eligible papers, and the organization of results. The Cochrane Collaboration method is detailed in the Cochrane Handbook for Systematic Reviews of Intervention [21]. Moreover, from the data abstraction phase onwards, PRISMA [22] guidelines will be followed. The PRISMA Statement provides an evidence-based 27-item checklist (e.g., focused on methodology) for reporting in systematic reviews and meta-analyses.

### Eligibility criteria

To determine the ways in which gamification has been used in intervention, a systematic approach will be conducted to retrieve relevant papers from the literature. Articles will be excluded from the review when: (i) they are not empirical studies; (ii) they are not written in Spanish, Portuguese or English languages; and (iii) they are not focused on gamification strategies. The following study characteristics will guide the process of inclusion and exclusion of articles. Study characteristics are described in Table 1.

**Table 1:** Description of study characteristics

Study characteristics	
Study design	Experimental studies (e.g., RCT’s) Quasi-experimental studies Observational studies when used to complement results from the experimental studies
Population	All medical conditions will be included as long as the intervention design uses gamification strategies Both adult and children will be considered as a criterion for inclusion
Interventions	Any intervention using gamification strategies will be considered Investigations focused on the efficacy or effectiveness of interventions (e.g., medical domain, educational domain) using gamification strategies will be included
Context	All countries and settings will be included. Specifically, clinical settings, educational settings, or rehabilitation settings

### Outcomes

The systematic review will allow authors to identify the gamification elements most used, the characteristics of the participants, the type and objectives of the intervention, the environment where the intervention is carried out, the assessment performed, and the effectiveness of these interventions.

### Information sources

This systematic literature review aims to identify articles containing information about interventions using gamification. For this purpose, searches will be undertaken in the databases as follows: Scopus, IEEE, Web of Science, MEDLINE, ERIC, and PsycINFO. The databases were included with the consideration of the scope. To complement this research, authors investigating the topic of gamification will be contacted and asked help to identify studies that are not selected in the research or studies in press. In addition, the references of the articles included will be checked with the same aim. This search will be conducted independently by two reviewers.

### Search strategy

This research will be carried out through the following expression: gamification OR “gam* element*” OR “gam* based” AND intervention. Database searches will not have a limit range regarding the years of publication. The search terms will be used for all fields (including title, abstract, keywords, and full text), and all results will be included. All studies will be coded independently by a member of the research team. Afterwards, a second reviewer will work independently to undertake a quality appraisal considering the inclusion criteria. If necessary, the disagreements will be resolved in a discussion with a third party. It should be noted that reviewers will not be blind to the titles of the papers or to their authors.

Finally, only studies meeting predetermined quality criteria will advance to data extraction.

### Data management

All records identified will be uploaded via EndNote X8 as a reference management software (Thomson Reuters, New York City, NY). This way, authors will easily identify any duplicates. Furthermore, this strategy will facilitate the exclusion ratios throughout the phases of the systematic review. Lastly, it will help build the diagram (i.e. PRISMA).

### Selection process

In the Identification and Screening phases to assess the eligibility for inclusion in the systematic review, the titles and abstracts will be screened by two reviewers independently. After the selection by abstract, the articles in full-text will be checked to select those eligible for the systematic review. When unpublished or ongoing articles are identified, authors will be contacted and invited to share the information to be included in the review.

### Data collection process

The two reviewers will independently extract the important information from the selected publications for the present review. The information extracted, mainly from the method and results sections, will analyze as follows: (i) type of intervention, (ii) participants, (iii) assessment, and (iv) results. Additionally, information about the gamification strategies used in each intervention, as well as relevant information of the intervention and assessment protocol will be collected. To minimize bias and improve the reliability of findings, paper scoring will be performed by the same researchers that analyzed and selected the paper and their relevant information. These two researchers will work independently; as a result of their work, papers found to meet the inclusion criteria will be eligible for the review. The results extracted will be summarized in accordance with the objectives of the study. Finally, the end report will be evaluated according to the PRISMA standards [22].

## Results

Expected results are as follows: a list of the gamification strategies used (e.g., leaderboards, rankings), data on the efficacy of the gamification strategies used, impact of the use of the gamification strategies on the therapeutic involvement, and outcomes of the intervention (e.g., stable insulin levels in diabetic patients).

Additionally, systematization of the intervention characteristics using gamification strategies (e.g., duration, context of the intervention) and the main domains in which these strategies are used (e.g., medical, educational or rehabilitation domain).

### Quality of evidence and risk of bias

Each study included will be scored by two investigators according to the quality criteria for articles. This process will rely upon the use of tools developed to assess the internal validity of the studies. For example, the Cochrane Effective Practice and Organization of Care Risk of Bias Tool, developed to assess risk of bias of experimental and quasi-experimental studies, will be used. Another example is the BEME quality indicators (e.g., A risk of bias assessment is included in the manuscript?) [23]. This quality assessment will be carried out by two reviewers, and disagreements will be resolved appealing to a third reviewer through team discussion. Only studies with a positive assessment will be included in the systematic review.

### Data synthesis

Authors will provide a narrative synthesis of the findings reported by the included studies. Results of the present investigation will be organized, among other aspects, according to the domain, type, and content of the intervention, the participants/patients’ characteristics, the main results, the methodological quality appraisal, and the risk of bias assessment. To summarize the results, all data extracted will be synthesized using tables and graphs. The results will be organized by chronological order and the authors of the papers will be contacted whenever information is missing. When possible, the current authors will provide summaries of the intervention effects for each study by calculating risk ratios (for dichotomous outcomes) or standardized mean differences (for continuous outcomes). The present authors anticipate that there will be limited scope for running a meta-analysis because of the lack of standardization across studies regarding the intervention protocols, measures used, and sample. Finally, the evidence of publication bias will be assessed.

### Data reporting

According to the PRISMA-P recommendations, the protocol was registered in the PROSPERO database. The systematic review will follow Cochrane’s recommendations and will be reported based on PRISMA guidelines.

## Discussion

This review will provide a synthesis of the extant literature on interventions using gamification’ strategies. Moreover, the impact of using distinct elements of gamification on outcomes will also be examined. This review will be conducted based on strict and stringent guidelines that assures the quality of the review. Based on the results, current authors will be able to systematize the body of knowledge on this domain and provide clear guidelines for practitioners interested in intervening using gamification strategies. In addition, lines for future research likely to address gaps in the literature will be suggested. Furthermore, we believe that faculty and practitioners would benefit from learning about a set of gamification strategies that are efficacious irrespective of the context or populations investigated. This corpus of knowledge is expected to be transferred and used to inform high quality interventions.

## Limitations

Regarding the limitations of the review, we can anticipate the publication bias as well as the language in which the article is published, since only articles written in English, Spanish and Portuguese are included.

## Additional file

**Additional file 1.** The PRISMA-P checklist used while drafting the protocol manuscript.

## Abbreviations

PRISMA: Preferred Reporting Items for Systematic Reviews and Meta-Analysis
PRISMA-P: Preferred Reporting Items for Systematic Reviews and Meta-Analysis-Protocols
RCT: Randomized Clinical Trials
